# Electrochemotherapy of skin metastases from malignant melanoma: a PRISMA-compliant systematic review

**DOI:** 10.1007/s10585-022-10180-9

**Published:** 2022-07-22

**Authors:** Martina Ferioli, Valentina Lancellotta, Anna Myriam Perrone, Alessandra Arcelli, Andrea Galuppi, Lidia Strigari, Milly Buwenge, Francesca De Terlizzi, Silvia Cammelli, Roberto Iezzi, Pierandrea De Iaco, Luca Tagliaferri, Alessio G. Morganti

**Affiliations:** 1 Radiation Oncology, IRCCS Azienda Ospedaliero-Universitaria di Bologna, DIMES, Alma Mater Studiorum - Bologna University, Via Albertoni 15, 40138 Bologna, Italy; 2 Dipartimento di Diagnostica per Immagini, Radioterapia Oncologica ed Ematologia, Fondazione Policlinico Universitario “A. Gemelli” IRCCS, UOC di Radioterapia Oncologica, Rome, Italy; 3 Oncologic Gynaecology, IRCCS Azienda Ospedaliero-Universitaria di Bologna, DIMEC, Alma Mater Studiorum - Bologna University, Bologna, Italy; 4 Medical Physics Unit, IRCCS Azienda Ospedaliero-Universitaria di Bologna, Bologna, Italy; 5 Scientific & Medical Department, IGEA S.p.A., Carpi (MO), Italy; 6 UOC Radiologia Diagnostica e Interventistica-Dipartimento di Diagnostica per Immagini, Radioterapia Oncologica ed Ematologia, Fondazione Policlinico Universitario “A. Gemelli” IRCCS, Rome, Italy; 7 Università Cattolica del Sacro Cuore, Rome, Italy; 8 Istituto di Radiologia, Ospedale Gemelli Molise, Campobasso, Italy

**Keywords:** Electrochemotherapy, Melanoma, Skin metastases, Systematic review, Local control

## Abstract

The main treatment of MM metastases are systemic therapies, surgery, limb perfusion, and intralesional talimogene laherparepvec. Electrochemotherapy (ECT) is potentially useful also due to the high response rates recorded in cancers of any histology. No randomized studies comparing ECT with other local therapies have been published on this topic. We analyzed the available evidence on efficacy and toxicity of ECT in this setting. PubMed, Scopus, and Cochrane databases were screened for paper about ECT on MM skin metastases. Data about tumor response, mainly in terms of overall response rate (ORR), toxicity (both for ECT alone and in combination with systemic treatments), local control (LC), and overall survival (OS) were collected. The methodological quality was assessed using a 20-item validated quality appraisal tool for case series. Overall, 18 studies were included in our analysis. In studies reporting “per patient” tumor response the pooled complete response (CR) was 35.7% (95%CI 26.0–46.0%), and the pooled ORR was 80.6% (95%CI 68.7–90.1%). Regarding “per lesion” response, the pooled CR was 53.5% (95%CI 42.1–64.7%) and the pooled ORR was 77.0% (95%CI 56.0–92.6%). One-year LC rate was 80%, and 1-year OS was 67–86.2%. Pain (24.2–92.0%) and erythema (16.6–42.0%) were the most frequent toxicities. Two studies reported 29.2% and 41.6% incidence of necrosis. ECT is effective in terms of tumor response and tolerated in patients with skin metastases from MM, albeit with a wide variability of reported results. Therefore, prospective trials in this setting are warranted.

## Introduction

The incidence of malignant melanoma (MM) in Europe is 150.627 new cases per year with a mortality of 26.360 deaths per year [1]. Around 2–20% of patients with advanced MM develop skin and/or subcutaneous metastases [2]. The main available treatment options of MM metastases are systemic therapies, surgery, limb perfusion, and intralesional talimogene laherparepvec (T-VEC) [3, 4].

Electrochemotherapy (ECT) is a well-established local therapy of primary and metastatic superficial tumors, whose efficacy was demonstrated by several clinical studies showing approximately 80% overall response rates (ORR) [5]. In addition, international operating procedures are available for ECT [5, 6]. Skin metastases with any histologic type can be treated with ECT. In fact, ECT acts trough transmission of electric pulses by creating transient cell membrane pores increasing drugs concentration and cytotoxic effect at intracellular level. Some clinical trials showed that bleomycin (BLM) and cisplatin (CDDP) are the most effective chemotherapy drugs when combined with electrical pulses ECT [6, 7]. Therefore, both are used during ECT. Moreover, electrical pulses lead to vasoconstriction thus resulting in drug trapping (“vascular lock”) and vascular disrupting effect [8]. Nowadays, ECT is mainly applied as a palliative treatment in order to improve patients’ quality of life, although in some cases it can allow for a prolonged local tumor control. Furthermore, ECT is increasingly being tested and used also in combination with systemic or other local treatments.

Considering the efficacy of ECT in other skin cancers [5, 9, 10], and the potential advantage of the hemostatic effect produced by the vascular lock in hemorrhagic lesions, some studies tested ECT in cutaneous MM metastases [11, 12]. However, no randomized study comparing ECT with other local therapies have been published on this topic.

Based on this background, the aim of this systematic review on melanoma skin metastases is to analyze the available evidence on efficacy and toxicity of ECT on skin metastases from MM.

## Materials and methods

Before starting the literature screening, the review was registered on the PROSPERO international register on January 4, 2021 [13]. The PRISMA (Preferred Reporting Items for Systematic Reviews and Meta-Analyses) guidelines were applied in the review process [14]. Primary endpoints were tumor response, mainly in terms of ORR, and toxicity of ECT alone or combined with other therapies. Secondary endpoints were local control (LC) and overall survival (OS). PubMed, Scopus, and Cochrane databases were screened by the authors and the reference lists of the included studies were also checked. The search strategy was as follows: ((electroporation[Title/Abstract]) OR (electrochemotherapy[Title/Abstract])) AND (melanoma[Title/Abstract]). Retrospective and prospective studies, case series, and clinical trials in the setting of MM skin metastases were included. Instead, we excluded case reports, paper reporting duplicate data, study protocols, papers where tumor response or toxicity were not reported or with tumor response and toxicity not reported separately from other tumors, systematic or narrative reviews, meta-analyses, letter-commentaries, editorials, planning studies, imaging studies, surveys, guidelines, recommendations and papers published not in English.

### Study selection

Two authors (MF, AA) independently assessed the literature articles, selected papers at title/abstract level, and removed duplicate. The full text of potentially eligible articles was analyzed. In case of disagreement, a third author (AMP) was involved in the final decision.

### Data extraction

Data were collected in an excel spreadsheet. The extracted data included: ECT characteristics (drug, route of administration, number of treatments, number and size of treated metastases), previous or concurrent treatments and their characteristics, tumor response after first ECT course in term of complete response (CR), partial response (PR), stable disease (SD), progressive disease (PD) and ORR, and toxicity. Two authors (MF, AA) independently extracted data and disagreement between individual judgments was solved by a third author (AMP).

### Quality assessment

Methodological quality was assessed using a 20-item validated quality appraisal tool for case series [15], by two authors (AG, MB), independently. Disagreements were solved by a third author (SC). Quality judgments for each item had a binary determination (“yes” or “not/not reported”) regarding study objectives, clear population description, interventions and co-interventions, outcome measures, statistical analysis, results, conclusions, and competing interests. A study with at least 15 “yes” responses was considered of acceptable quality.

### Statistical analysis

Statistical analysis was carried out using the MedCalc statistical software (version 15.2.2, MedCalc Software bvba, Ostend, Belgium). All tests were two-sided. The I2 statistic was used to quantify statistical heterogeneity (high heterogeneity level: > 50%). The latter was tried out with the Q2 test. Statistical significance was considered as p < 0.05, except when investigating heterogeneity among studies (p < 0.1). In case of heterogeneity among selected studies, rates and proportions were pooled using a random-effects model. A fixed-effect model was used in other cases. The dependent variables were modeled on the logit (log-odds) scale, converted back to percentages, and then presented as point estimates and 95%CI.

## Results

**Fig. 1:**
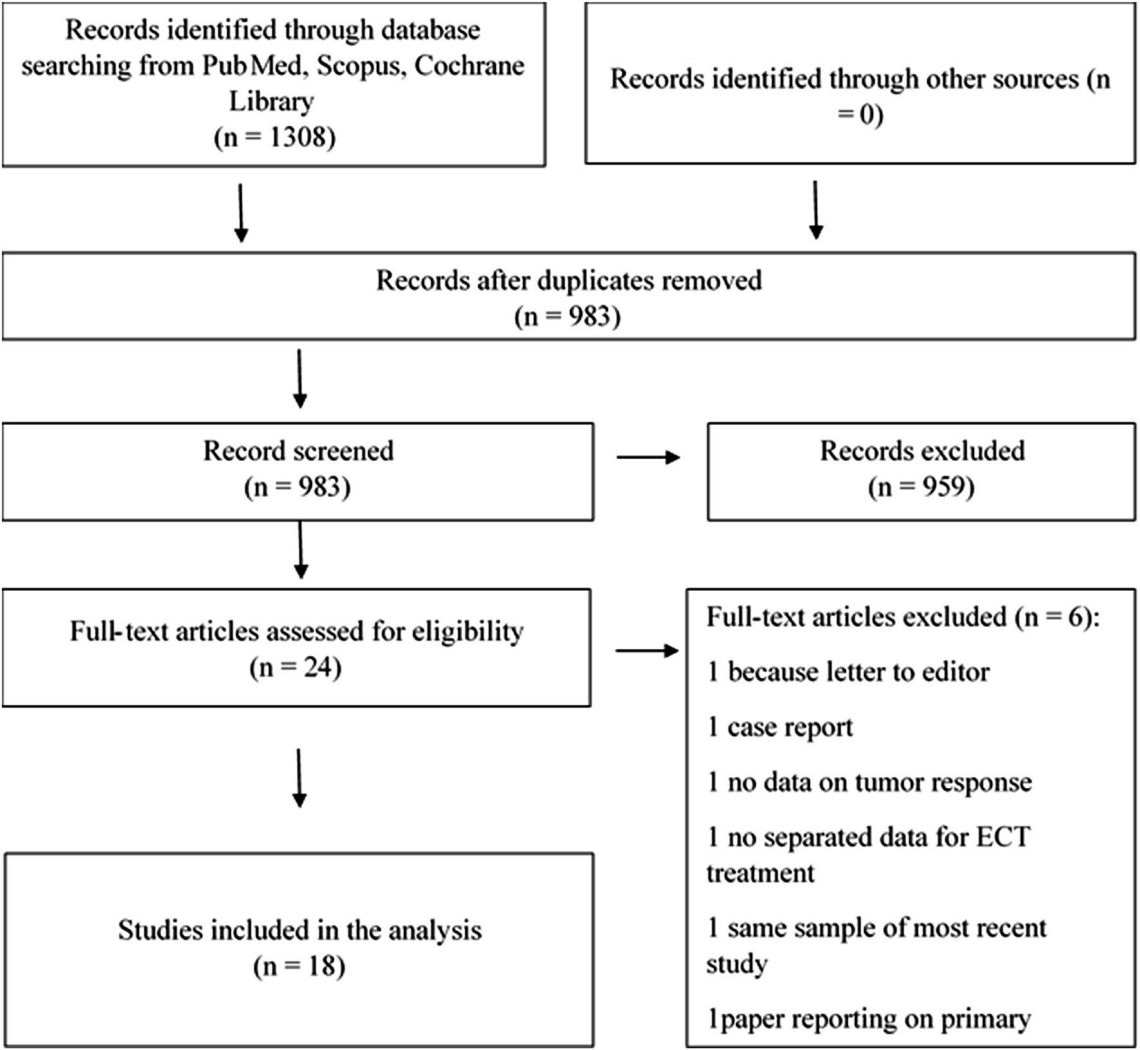
PRISMA Flow-chart

The search results are shown in the PRISMA flow-chart (Fig. 1). After duplicate removing, 983 articles were evaluated, and 24 full-text articles were selected. Six of them were excluded from the final analysis for the following reasons: one letter to editor, one case report, one with no data on tumor response, one without separated data on ECT, one reporting on the same patients population of a more recent study by the same authors, and one reporting on primary MM. Seven studies had a retrospective design [16–22], ten were prospective [11, 12, 23–30] and one was a retrospective-prospective analysis [31]. Six studies were published before ESOPE guidelines were issued in 2006 [16–18, 23, 24, 26]. Studies’ characteristics are described in Tables 1 and 2.

**Table 1:** Patients and treatment characteristics

Authors/year	Study design	No. patients treated/ evaluable	No lesions treated/ evaluable	Lesions per patient	Drug	Route	Electrode	Anesthesia	No ECT courses	Lesions size	Previous therapies	Concurrent therapies	Site of lesions
Rudolf Z. et al., 1995	Retrospective	2/2	24/24	Range 1–13	BLM	IV	Long needles	LA	1–3	Range 16–2915^a^	Surgery, CHT, IFN, RT	None	Extremities
Glass LF. et al., 1996	Retrospective	5/5	23/23	NR	BLM	IT	Plate/hexagonal	LA	1	NR	NR	NR	NR
Rols MP. et al., 2000	Retrospective	4/4	55/55	Range 10–22	BLM	IV	Plate	GA/LR	1	Range 2–35^b^	Surgery, CHT, RT	None	Trunk, extremities
Sersa G. et al., 2000	Phase I-II	9/9	27/27	NR	CDDP	IV	Linear	LA	1	Mean 1010^a^	NR	Vinblastine, lomustine, IFN alfa	NR
Sersa G. et al., 2000	Phase II	10/10	133^c^	Range 1–44	CDDP	IT	Linear	LA	1	Median 61 (range 2–39,270)^a^	surgery, CHT, IFN	None	NR
Byrne CM. et al., 2005	Phase II randomized	19/15	46/36^d^	Range 2–4	BLM	IT	Linear	LA	1	Range 3-50^b^	Surgery, ILP	None	Extremities abdomen, neck
Gaudy C. et al., 2006	Randomized controlled	12/10	54/40^e^	Range 2–12	BLM	IT	Hexagonal	LA	1	Median 10 (range 3–26)^b^	NR	CHT in 8 pts (66%)	NR
Quaglino C. et al., 2008	Prospective	14/14	233/233	Mean 10	BLM	IV	Plate/hexagonal/linear	GA	1–3	Range 2-75^b^	CHT, ILP	NR	Extremities
Kis E. et al., 2011	Retrospective	9/9	158/158	Mean 17.5 (range 1–62)	BLM	IV	Hexagonal/linear	GA	1	Mean 1.47 (0.5–5.6 cm)^b^	Surgery, CHT, IFN, RT	None	Trunk, extremities
Campana LG. et al., 2012	Prospective	85/85	894/894	Median 11 (1-> 50)	BLM	IV-IT	NR	LA-GA-S	1–6 (median 3 courses)	Median 24 (range 3–75)^b^	CHT, IFN, RT, ILP	None	Trunk, extremities
Ricotti F. et al., 2014	Prospective	30/30	654/654	Median 21.8 (range 4–54)	BLM	IV	NR	GA	1–2	10 cm^2^	NR	None	NR
Mir-Bonafè JM. et al., 2015	Retrospective –prospective	31/31	NR	NR	BLM	IV	Hexagonal	NR	1–3	NR	surgery, CHT, ILP	None	HN,trunk,extremities
Caracò C. et al., 2015	Retrospective	89/89	NR	NR	BLM	IV	Plate/hexagonal	GA-LR	1–6	median 12 (range 2–35)^b^	surgery	None	HN,trunk,extremities
Mozzillo N. et al., 2015	Retrospective	15/15	NR	NR	BLM	IV	NR	NR	NR	NR	CHT	Ipilimumab	Skin
Hribernik A. et al., 2016	Retrospective	5/5	111/111	Range 1–80	BLM-CDDP	IV-IT	Plate	NR	1	Median 1.5 cm (range 0.8–3)^b^	Surgery, IFN	None	Trunk, extremities
Heppt M. et al., 2016	Retrospective	33/33	NR	NR	BLM	IV-IT	Hexagonal/linear	GA-LA	NR	NR	CHT, immuno-targeted therapy, RT, ILP	Concurrent immunotherapy^g^ within 4 weeks	HN,trunk,extremities
Tomassini GM. et al., 2016	Prospective	6/6	69/9^f^	NR	BLM	IV	Hexagonal/linear/finger	S	1	Range 2–260^b^	NR	NR	Trunk, extremities
Kunte C. et al., 2017	Prospective	151/114	506/394	Median 3 (range 1–6)	BLM	IV-IT	Plate/ hexagonal/linear	GA-LA	1–4	Median 9 (range < 5–>30)^b^	Surgery, CHT, RT, ILP	NR	HN,trunk,extremities

*BLM* Bleomycin, *CDDP* cisplatin, *CHT* chemotherapy, *ECT* Electrochemotherapy, *EP* electroporation, *IFN* interferon, *ILP* isolated limb perfusion, *IT* intratumoral;IV: intravenous; *LA* local anesthesia, *LR* loco-regional anesthesia, *NR* not reported, *RT* radiotherapy, *GA* general anesthesia, *S* sedation

^a^Lesions dimension reported as volume (mm^3^ unless otherwise reported)

^b^Lesions dimension reported as diameter (mm unless otherwise reported)

^c^82 lesions treated with ECT, 27 with CDDP, 2 with EP, and 22 were controls

^d^18 lesions treated with ECT vs. 18 treated with BLM alone

^e^30 lesions treated with ECT and 24 with BLM alone

^f^9 target lesions and the other considered “no target lesions”

^g^Ipilimumab-pembrolizumab-nivolumab

**Table 2:** Tumor response and toxicity

Author/year	Time of response evaluation	Follow-up duration, months-median (range)	CR (%)	PR (%)	SD (%)	PD (%)	ORR (%)	Response evaluation (lesions or patients)	Scale	Toxicity
Rudolf Z. et al., 1995	4 weeks	NR	92	0	4	4	92	Lesions	WHO	Erythema, muscle spasm, local pain
Glass LF. et al., 1996	12 weeks	NR	78	17	4	0	96	Lesions	NR	Erythema and edema
Rols MP. et al., 2000	NR	NR	9	81	NR	NR	90	Lesions	NR	Erythema, edema, superficial necrosis, hyperthermia,
Sersa G. et al., 2000	4 weeks	NR	11	37	41	11	48	Lesions	WHO	Erythema
Sersa G. et al., 2000	4 weeks	35 (5–124)	68	10	15	7	78	Lesions	WHO	Erythema and edema
Byrne CM. et al., 2005	12 weeks	M: 21	72	5	18	5	77	Lesions	WHO	Pain, muscle spasm
Gaudy C. et al., 2006	4 weeks/ 12 weeks	24	74–64	13–18	NR	NR	87–82	Lesions	WHO	Pain 75%, muscle spasm 25%, erythema 16.6%, necrosis 41.6%
Quaglino C. et al., 2008	8 weeks	21	50/58	43/34	7/8	0	93/92	Lesions/patients	WHO	Erythema 21.4%, pain 0%
Kis E. et al., 2011	8 weeks	195 days (60–358)	23	39	30	8	62	Lesions	WHO	Erythema, edema
Campana LG. et al., 2012	4 weeks	26 (6–47)	44^a^/48	0/46	55/4	1/2	44/94	Lesions/patients	RECIST	Pain 92%, syncope 4.7%, nausea 9.4%, fever 4.7%, skin G3 18%
Ricotti F. et al., 2014	4 weeks	20	67.3^b^/20	32.7/80			100/100	Lesions/patients	WHO	NR
Mir-Bonafè JM. et al., 2015	4 weeks	NR	23	49	0	28	72	Patients	RECIST	Ulceration and infection (25.8%), pain, edema, erythema, nausea, vomiting
Caracò C. et al., 2015	12 weeks	27.5 (6–67)	48.3	38.2			67.5	Patients	WHO	Pain 37%, myalgia 13.5%, necrosis 29.2%
Mozzillo N. et al., 2015	4 weeks	NR	27	40	0	33	67	Patients	WHO	Pruritus 80%
Hribernik A. et al., 2016	4 weeks	NR	85					Lesion	WHO	None
Heppt M. et al., 2016	Median 12 weeks (range 4–32)	9	15.2	51.5	9.1	24.2	66.7	Patients	RECIST	Ulceration 45.5%, erythema 42.4%, infection 30.3%, pain 24.2%, nausea 9.%% ≥ G3)
Tomassini GM. et al., 2016	8 weeks	NR	33.3	0.0	44.4	22.3	33.3	Lesions^c^	RECIST	NR
Kunte C. et al., 2017	8 weeks	116 days	58/48	20/25	20/26	2/3	78/74	Lesions/patients	RECIST	Skin toxicity 50% (G3 in 2 pts), nausea 4%, lymphedema 4%, flu like symptoms 5%, pain 39%

*CR* complete response, *M* mean, *NR* not reported, *ORR* objective response rate, *PD* progression disease, *PR* partial response, *RECIST* response evaluation criteria in solid tumors, *SD* stable disease, WHO World Health Organization

^a^78% after second course in previous PR lesions

^b^After first course, after the second course the CR was 89% of lesions

^c^Response evaluated on the nine target lesions; all non-target lesions were classified as SD

### Patients and tumors characteristics

Overall, as showed in Tables 1 and 529 patients were treated with ECT on 2987 skin MM metastases. However, five papers did not report the number of treated metastases [19, 20, 22, 29, 31]. Most lesions were in the trunk and extremities. The size of the treated metastases was reported heterogeneously: some authors reported the volume [11, 16, 23, 24], others the diameter [12, 18, 19, 21, 25–30], while four papers did not describe this data [17, 20, 22, 31]. Looking at the diameter, the range was 2–260 mm. Most articles also reported the number of lesions per patient, with figures ranging between one and 80 lesions. Furthermore, before ECT, most patients included in the analyzed papers underwent chemotherapy, surgery and interferon. Radiotherapy was reported as previous treatment in six studies [12, 16, 18, 22, 28, 30] with available data on the number of previously irradiated patients on the same area of ECT. One paper also described the toxicity in this patients subgroup [12].

### Treatment characteristics

ECT was based on BLM in 15 studies [11, 12, 16–20, 22, 25–31], on CDDP in two studies [23, 24] and on both drugs in one study [21], with intravenous [11, 12, 16, 18–22, 24, 27–31] or intratumoral [12, 17, 21–23, 25, 26, 30] administration. The used electrodes were mainly linear [22–25, 27–30], hexagonal [17, 19, 22, 26–31] and plate [17–19, 21, 27, 30], while anesthesia was mostly local [12, 16, 17, 22–26, 30]or general [11, 12, 18, 19, 22, 27, 28, 30]. Overall, ECT courses ranged from one to six. Four authors reported that ECT was concurrent to chemotherapy [24, 26] or immunotherapy [20, 22]. In the others papers the lack of concurrent therapies was declared [11, 12, 16, 18, 19, 21, 23, 25, 28, 31] or this information was not available [17, 27, 29, 30].

### Outcomes

#### Local control

**Fig. 2:**
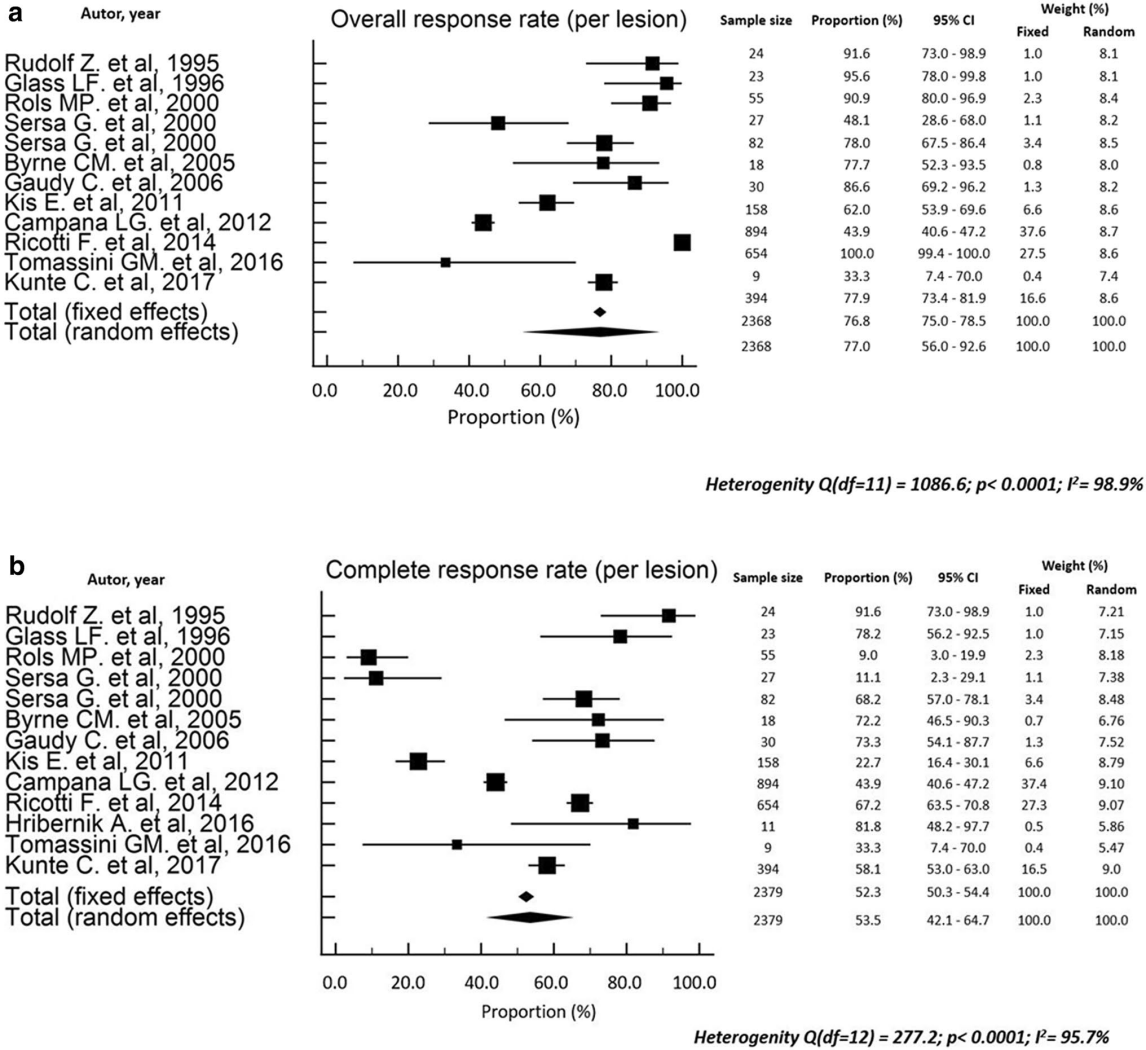
**a** Forest plot of the overall response rates reported on a per lesion basis. **b** Forest plot of the complete response rates reported on a per lesion basis

**Fig. 3:**
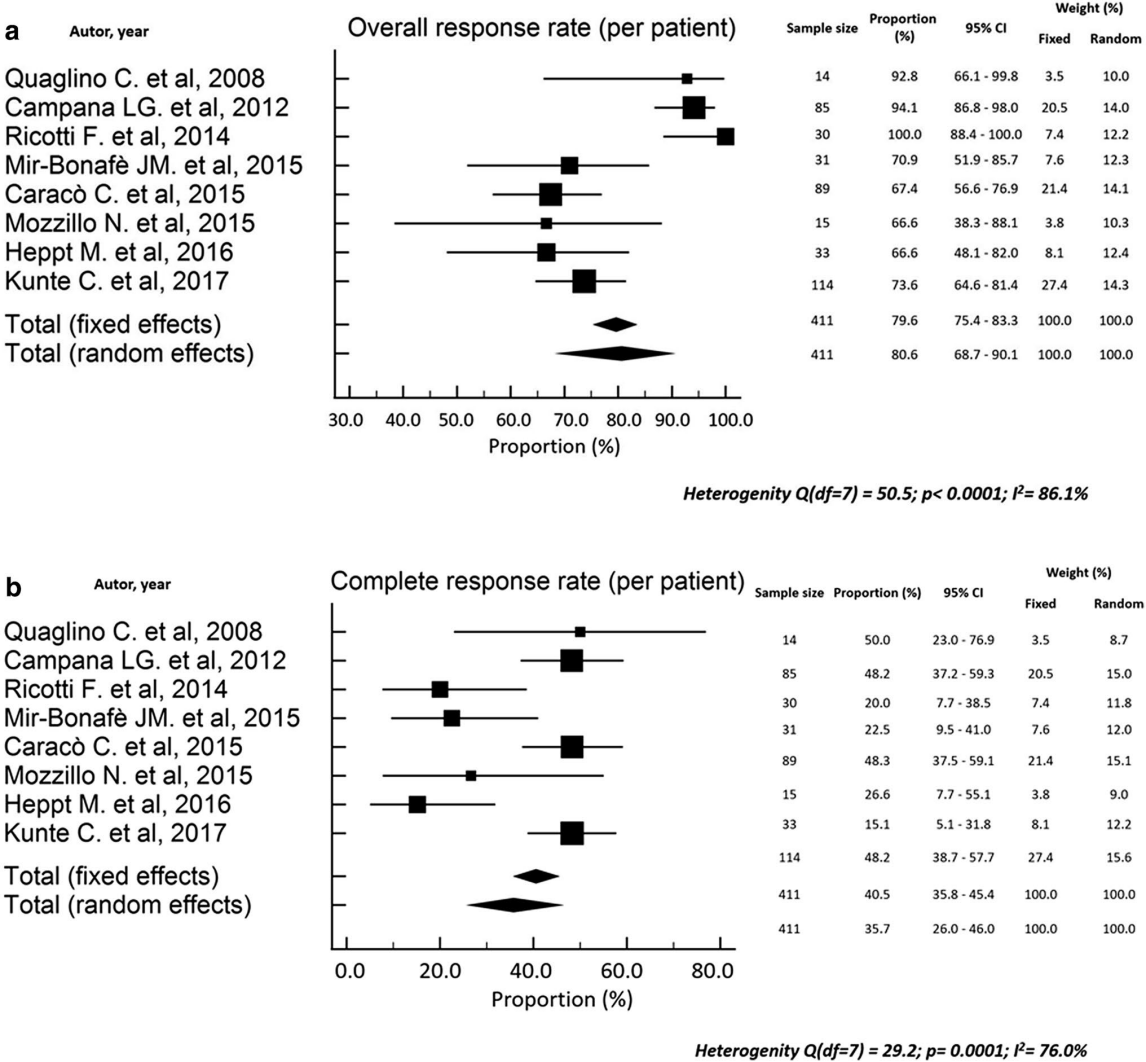
**a** Forest plot of the overall response rates reported on a per patient basis. **b **Forest plot of the complete response rates reported on a per patient basis

The clinical response was reported in terms of local tumor response (CR, PR, SD, PD, ORR) on a “per lesion”[16–18, 21, 23–26, 28, 29] or a “per patient” basis [19, 20, 22, 31] or both [11, 12, 27, 30], as shown in Table 2. In studies reporting “per lesion” tumor response, the ORR ranged between 33.3 and 100% (pooled rate: 77.0%; 95%CI 56.0–92.6; Fig. 2a), and the CR between 9 and 92% (pooled rate: 535%; 95%CI 42.1–64.7; Fig. 2b). Moreover, the “per patient” ORR ranged between 66.7 and 100% (pooled rate: 80.6%; 95%CI 68.7–90.1; Fig. 3a) and the CR between 15.2 and 50% (pooled rate: 35.7%; 95%CI 26.0–46.0; Fig. 3b). The timing of response evaluation after ECT was heterogeneous among studies but most assessments were performed four weeks after the first ECT course [11, 12, 16, 20, 21, 23, 24, 26, 31]. The Response Evaluation Criteria in Solid Tumors (RECIST) scale and the World Health Organization (WHO) criteria were used in five [12, 22, 29–31] and 11 [11, 16, 19–21, 23–28] papers, respectively, while two studies did not report the tumor response scoring system [17, 18]. Actuarial LC was reported in four studies [11, 12, 27, 30]. Three papers reported 72–87% 2-year LC, with 80% 1-year LC in one paper, and one study reported 86% LC rate after 200-days.

#### Survival outcomes

Other clinical outcomes were reported in some study: three papers analyzed OS and reported 67-86.2% 1-year OS [20, 30] and 15 months OS in patients treated with ECT *plus* concurrent anti-Programmed Death 1 (PD1) immunotherapy [22], respectively. PFS was reported by two authors [12, 22]. Campana et al. reported 87% 2-year local PFS [12] while Heppt et al. reported 2.5 months median PFS [22]. Melanoma specific survival was reported by Kunte et al.as 74% 1-year rate [30]. Only ten studies reported the follow-up duration [11, 12, 19, 22, 23, 25–28, 30], ranging between 116 days and 35 months.

#### Internal comparisons

Three studies compared ECT *versus* BLM- or CDDP-based intravenous chemotherapy alone [24–26] reporting significantly higher CR rates after ECT. Finally, two authors reported higher LC rates in smallest lesions, especially those < 3 cm [12, 30]. Despite ECT was mostly used as palliative treatment, only one study reported the results in terms of symptoms relief [29], showing a reduction in bleeding and pain, while no paper analyzed the Quality of Life.

### Toxicity

Seven papers reported only which toxicity was the most frequent [16–18, 23–25, 28] while the others reported the specific rates [12, 19, 20, 22, 26, 27, 30, 31]. Pain and erythema were the most common side effects. More specifically, pain was reported by 24.2–92.0% of patients and erythema was recorded in 16.6–42.0% of subjects. Two studies [19, 26] reported a non-negligible incidence of necrosis (41.6% and 29.2%, respectively). Finally, some authors reported variable rates of infections, ulcerations, muscle spasms, and nausea, as shown in Table 2.

### Quality assessment

Only seven studies were of acceptable quality after evaluation with the case series quality appraisal checklist [12, 23, 25–28, 30]. In fact, all authors reported the number of patients but four studies did not report the number of treated lesions [19, 20, 22, 31]. Four studies did not report the metastases size [17, 20, 22, 31] and the lesions site was not reported in five studies [11, 17, 23, 24, 26]. Previous therapies were described by most authors [12, 16, 18–23, 25, 27, 28, 30, 31], but often as general information while only few authors specifically reported previous local treatments on ECT-treated lesions [12, 16, 18, 22, 28, 30]. Finally, the tumor response was reported on a “per lesion” or “per patient” basis by most authors with only four papers reporting both [11, 12, 27, 30].

## Discussion

In our systematic review on ECT in skin MM we recorded 53.5% and 77% pooled complete and overall response rates on a per lesion basis, respectively. Most frequently reported toxicities were pain and erythema which were reported as mild in most patients. Therefore, based on the available evidence, ECT can be considered well tolerated and effective in terms of tumor response in this setting, albeit with a wide variability of the reported results [9, 10, 32, 33]. Finally, the pooled rates reported in this analysis can serve as a benchmark for further studies aimed at improving current outcomes by optimizing treatment techniques and patient selection, as well as improving treatments combinations.

Some authors suggested an immunostimulating effect of ECT [34, 35], and therefore a possible synergistic effect of ECT and immunotherapy [3]. Therefore, considering the efficacy of immunotherapy in MM, some studies included in our analysis tested this combined modality treatment [20, 22].

Nevertheless, in our analysis we did not record improved results in series of combined ECT *plus* immunotherapy [20–22]. In fact, the ORR rate after ECT alone was 67.5–100.0% [11, 12, 19, 27, 30, 31]while after ECT *plus* immunotherapy was 66.7–67.0% [20, 22]. It should be noted that in some series on combined modality treatment the 1-year OS rates were relatively high (86.2%) [20]. However, a comparison with ECT alone in terms of OS was not feasible being this endpoint not reported in most of the latter papers [11, 12, 19, 27, 31].Comparing immunotherapy alone or combined with local treatments, Theurich et al. reported the outcomes after Ipilimumab *versus* Ipilimumab *plus* local therapies (radiotherapy, ECT). The addition of local treatment to ipilimumab significantly prolonged OS (median: 93 *versus* 42 weeks, p: 0.0028) [36]. However, only four out of 45 enrolled patients were treated with ECT. Finally, Campana et al., in their recent retrospective analysis, showed how Pembrolizumab combined to ECT in melanoma patients (stage IIIC-IV) improves 1-year PFS and OS (p = 0.034 and p = 0.006, respectively) [37].

In our analysis no series reported on combined RT *plus* ECT. However, Kunte et al. reported worse LC rates in previously irradiated metastases [30].

Among studies reporting tumor response on a “per lesion” basis, we recorded a wide variability both in terms of CR (9.0–92.0%) and ORR (33.3–100.0%). Rols et al. [18] and Rudolf et al. [16] reported the lowest and the highest CR rate, respectively. Both authors treated few patients (four and two patients with 55 and 24 lesions, respectively) but the first used a plate electrode, usually able to treat only the exophytic region of the skin lesions, while the other used the long needles electrodes, able to better treat the whole tumor volume. In terms of ORR rate, Tomassini et al. and Ricotti et al. reported 33.3% [29] and 100.0% rates [11], respectively. Beyond the different parameters used to evaluate the lesions size in the two studies (diameter vs. area), it can be observed that the maximum tumor size in the first series were clearly larger than the second one (260 mm vs. 10 cm^2^) and this might explain the different response rates.

Smaller differences were recorded regarding the response rates assessed on a “per patient” basis. In fact, the CR rates ranged between 15.2% and 50.0% and the ORR rates between 66.7% and 100.0%. Heppt et al. reported the worst CR and ORR rates, but they did not describe either the lesions number or the tumors size. Therefore, we cannot assess whether the poor local efficacy is attributable to these factors. However, it can be noted that nearly 30% of MM metastases were in previously irradiated areas and this may have had a negative effect on ECT efficacy due to reduced tissue perfusion and its consequences on the electrical pulses transmission [22]. Furthermore, the drug administration route was both intravenous and intratumoral. Instead, the authors who reported the best response rates [11, 27] used only the intravenous route, which is known to be more effective especially in larger lesions [5].

Overall, pain and erythema were the most common toxicities. Unfortunately, different scoring systems and lack of information on incidence rates make it difficult to assess frequency, severity, and predictors of adverse events in many studies. However, it should be noted that necrosis and ulceration were more frequent in patients treated with hexagonal electrodes [22, 26, 31] and in subjects undergoing concurrent chemotherapy [26] or immunotherapy [22]. Since the hexagonal electrode can provide a higher number of electrical pulses and with a higher voltage, compared to other electrodes, it could have favored ulceration and pain [5].

As previously mentioned, several treatment options are now available for MM metastases. Therefore, a comparison of the results of the latter with those of the ECT would be useful.

The ORR was 10–20% using Dacarbazine-based therapy in patients with metastatic MM, a figure lower compared to the rates recorded after ECT [38]. Similarly, a phase II study on metastatic MM treated with T-VEC, the first and only oncolytic virus approved by the FDA for the treatment of MM, reported 26% ORR, still lower than ECT results [39]. However, results from phase II and III clinical trials as well as real world data showed 26.4% [40], 39%, and 18% [41] ORR, CR, and PR rates after T-VEC therapy, respectively, which appears to be able to stimulate local and systemic immune responses similar to ECT.

It is difficult to identify studies separately reporting the response of skin lesions to immunotherapy, as treated patients often have multi-organ metastases. However, it can be observed that in the CA184-007, CA184-008, and CA184-022 trials the ORR was 40% [42]. Finally, in a phase III trial based on combined Ipilimumab *plus* Dacarbazine, the ORR was 50% [43]. Therefore, it would be interesting to analyse immunotherapy efficacy in patients affected only by cutaneous metastases. Regarding the comparison between ECT and other local therapies, Moreno-Ramirez et al. [44], in their review on limb perfusion of in-transit MM lesions, reported 90% ORR. These results are similar to those of ECT. However, it should be noted that limb perfusion has site limitations being this treatment mainly directed to metastases in the extremities. Byers et al. [45] reported on in-transit MM metastases treated with intralesional therapy (mainlyIL-2). The ORR was 80.5%, again similar to ECT.

Therefore, the comparison among our analysis and available literature data shows that ECT local response rates are superior or at least like those of other treatments, without site limitations as in the case of limb perfusion.

In terms of predictors of ORR after ECT, we compared, based on previous irradiation, the studies included in our review in terms of tumor response. In papers including [12, 22, 28, 30] or not including [19–21, 23, 24, 27, 31] previously irradiated patients, the ORR was 44–78% and 67–93%, respectively, suggesting a negative impact of radiotherapy delivered prior to ECT. Furthermore, Kunte et al. [30] recorded an independent negative impact, on multivariate analysis, of prior irradiation on tumor response. Finally, another analysis, however also including patients with non-melanoma tumors, confirmed the lower CR rate in patients with previous radiotherapy (59% versus 71%) [33].

Our analysis has some limitations: eight studies were retrospective [16–22, 31], ECT was based on different drugs, with different doses and routes of administration, the number of lesions and patients was reported in different ways, the tumor size was assessed and reported based on various parameters, five studies did not report previous therapies [11, 17, 24, 26, 29], data on previous radiotherapy was largely missing, and toxicity was described only narratively in most studies [16–18, 23–25, 28]. Moreover, after quality assessment, less than half of the studies were rated as sufficient. Furthermore, papers published prior to the ESOPE guidelines, when ECT was still considered an experimental treatment, were also included in the analysis [16–18, 23, 24, 26]. However, we chose to avoid chronological limitations in the inclusion criteria in order to provide the broadest overview of the available evidence. Finally, the Quality of Life was not analyzed, despite the palliative treatment setting.

However, our results are confirmed by the findings of a very recent publication by Petrelli et al. [46], who analyzed the available publications in order to evaluate ORR, LC, and OS after ECT in cutaneous MM. The results of their report are very similar to the findings of our analysis. Indeed, ORR, 2-year LC, and 1-year OS were 77.0% and 77.6%, 72–87% and 72–74%, and 67–86% and 67–89% in our and in Petrelli’s et al. analyses, respectively. Therefore the two review are in agreement in suggesting the local efficacy and long-lasting LC after ECT in this setting.

## Conclusions

The results of our analysis suggest that ECT may be considered a treatment option in patients with MM skin metastases. Considering the low grade of available evidence and the need to individualize treatment, especially in the metastatic setting, these patients should ideally be managed by multidisciplinary teams including dermatologists, medical oncologists, and radiation oncologists.

However, considering the promising local response rates, further studies on ECT in this setting are warranted. These trials should aim at: (i) careful assessment of long-term results, both in terms of local control and side effects; (ii) evaluation of the impact of ECT on symptom relief and quality of life; (iii) definition of the best combinations with systemic therapies, in particular with immunotherapy, both in terms of drugs or drug combinations and in terms of timing of the therapeutic sequence.

## Data Availability

All data analysed during this study are included in this published article.
